# Special Issue: Molecular Mechanisms and Pathophysiology of Myocardial Disease

**DOI:** 10.3390/ijms26199400

**Published:** 2025-09-26

**Authors:** Inna P. Gladysheva, Ryan D. Sullivan

**Affiliations:** 1 Department of Internal Medicine, Translational Cardiovascular Research Center, College of Medicine-Phoenix, University of Arizona, Phoenix, AZ 85004, USA; ryansullivan@arizona.edu; 2 Clinical Translational Sciences (CTS), Bio5 Institution, University of Arizona, Tucson, AZ 85721, USA

According to the statistical updates from the Centers for Disease Control and Prevention and American Heart Association in conjunction with the National Institutes of Health [1], heart disease remains to be a leading cause of death, and heart failure (HF) is a leading cause of hospitalizations in the USA and globally, despite advancements in understanding and management.

Myocardial disease presents a heterogenous complex spectrum of diverse cardiac muscle pathologies, including cardiomyopathies (dilated, hypertrophic, restrictive, and other forms), acute or chronic ischemic cardiac disease (reduced blood flow and myocardial infarction), arrhythmias (irregular heartbeats), cardiac valvular dysfunction (heart valve disease), and myocarditis (cardiac inflammation), which may independently occur, contribute to each other, or co-exist with non-cardia comorbidities, including diabetic, metabolic, infectious hepatic, renal, and others. These cardiac pathologies are characterized by a range of progressive structural (remodeling and fibrosis) and functional abnormalities of the heart muscle, which often result in symptomatic HF with pleural and systemic salt and water retention (edema/congestion) [2,3], leading to poor quality of life, disability, and premature sudden death. As the population ages worldwide, there is a dramatic increase in myocardial disease, with a higher prevalence and incidence of HF in older adults [1,4,5,6].

Over the past 5 years, new classes of pharmacotherapies, including mineralocorticoid receptor antagonists, angiotensin receptor–neprilysin inhibitor (ARNI), sodium–glucose cotransporter-2 inhibitors, colchicine, soluble guanylate cyclase stimulators, and glucagon-like peptide-1 receptor agonists, have been successfully implicated in HF management, reducing the risk of cardiovascular death and HF hospitalization [7,8,9,10,11,12,13,14,15,16]. Still, the incidences of HF are growing [17,18], and symptomatic HF patients have no cure, except for a temporary abatement of symptoms by heart transplantation. Obviously, novel scientific insights are required for a comprehensive understanding of the complex pathophysiology and mechanisms of myocardial disease to promote advanced therapeutic approaches.

This Special Issue comprises the original research articles and reviews demonstrating progress in clinical and pre-clinical research of the latest research on the pathophysiology and molecular mechanisms of myocardial disease, which may lead to innovative approaches that are critical for disease prevention and for the development of novel therapies.

Aortic valve (AV) stenosis, or a narrowing of the aortic valve opening, leading to blood flow obstruction to the rest of the body, is one of the most common and serious types of heart valve disease. Severe forms of AV stenosis affect about 2–9% of people over the age of 75. Taiki Tojo and Minako Yamaoka-Tojo [19] identified key molecular mechanisms and hub genes involved in the progression of AV stenosis using bioinformatic analysis of publicly available gene expression datasets from human aortic stenosis and normal AV tissue combined with analyses of the protein–protein interactions of the differentially expressed genes. Specifically, this study revealed the positive regulation of cell adhesion, leukocyte-mediated immunity, response to hormones, cytokine signaling in the immune system, lymphocyte activation, and growth-hormone-receptor-signaling pathways and suggested potential biomarkers and therapeutic targets for aortic stenosis, laying the foundation for future experimental studies.

The healthy human and animal heart requires proper energy metabolism to effectively maintain blood flow throughout the body by rhythmic coordinated cardiac muscle contraction (systole) and relaxation (diastole). The mechanisms of cardiac metabolism and its impact on cardiac pathologies have been studied for decades [20,21]. Alfredo Caturano et al. [22] provide an overview of the insulin–heart axis, focusing on insulin’s multifaceted impact on cardiac function. The authors introduce and discuss the insulin-driven molecular signaling mechanisms altering glucose and fatty acid metabolism in cardiomyocytes or cardiac muscle cells that enable the pumping action of the heart and highlight its role in cardiac growth and other physiological processes. The authors focus on how the insulin resistance disrupts cardiac glucose and lipid metabolism, causes autonomic dysfunction, oxidative stress, chronic inflammation, and activation of the renin–angiotensin–aldosterone system, resulting in the development of diabetic cardiomyopathy with cardiac diastolic and/or systolic dysfunction and hypertrophic/fibrotic remodeling that may progress to symptomatic HF. Together with another review from the same team [23], this work also provides a summary of current therapies for diabetic cardiomyopathy and an overview of future research to advance the therapeutic options for insulin-resistant populations.

Myocardial injury and HF are associated with hypoxia, a condition characterized by acute or chronic reduced oxygen levels in the body’s tissues due to physiological (reduced oxygen content at high altitude) or pathological (including myocardial ischemia and HF) causes. The master regulator of the cellular response to hypoxia is hypoxia-inducible factor 1 alpha (HIF1α) [24]. In HF, altered signaling of HIF-1α in peripheral blood mononuclear cells (PBMCs) impaired the transcription of genes involved in angiogenesis, metabolism, and inflammation]. Although PBMCs do not transport oxygen, their metabolic processes and mitochondrial function appear to be highly sensitive to oxygen levels and are altered during hypoxia. Marianne Riou et al. [25] reported that severe acute hypoxia impairs PBMCs’ mitochondrial respiration and reactive oxygen species production in 13 prospectively enrolled healthy volunteers exposed to controlled oxygen reduction. Specifically, in human PBMCs, severe acute hypoxia impairs complex II respiration and is inversely correlated with the succinate receptor SUCNR1, HIF-1α, and markers of inflammation. Overall, the study outcomes improve our understanding of hypoxia pathophysiology in humans, encouraging further research to investigate whether modulation of complex II activity is involved in the pathogenesis of HF.

Animal models play a vital role in advancing cardiac research [26,27]. They are essential to study the cellular and molecular mechanisms of progressive cardiac pathologies and for preclinical testing of new therapies and devices. Mice and rats are the most economical and widely used animal models, with a high reproduction rate and well-understood genetics, which enable surgical, chemical, genetic (permanent or inducible overexpression or knockout), and lineage tracing manipulations to replicate specific human cardiac pathologies, including cardiomyopathies, myocardial infarction, and heart failure [28,29,30,31].

Cardiac fibrosis, or the excessive diffuse deposition of extracellular matrix in the cardiac interstitium, is a common pathophysiologic manifestation of most myocardial diseases [32]. In this Special Issue, two original research papers and one review advance our understanding of this pathology, presenting studies implicating mouse models of cardiac fibrosis.

Mohammed Mimouni and his colleagues review the progress over the last 15 years in the development of models to study endothelial-to-mesenchymal transition (EndoMT) in cardiovascular diseases, specifically focusing on cardiac fibrosis, discussing their relevance and specific advantages and limitations [33]. The authors overview is both in vitro (including cytokine-, hypoxia-, and high-glucose-based) and in vivo (mouse endothelial-specific inducible Cre/Dre-lox lineage recombinase tracking systems, in which they could trace cardiac endothelial cells expressing or not expressing fibroblast markers to investigate EndoMT). Strikingly, the advanced genetic tracing systems demonstrated that cardiac endothelial cells transdifferentiate into myofibroblasts and transiently activate mesenchymal genes only during embryonic mouse heart development, but not in adult injured hearts, and that resident fibroblasts are the primary source of myofibroblasts in cardiac fibrosis [34].

Chagas disease (CD) is endemic to Mexico, Central America, and South America, but affects approximately 7 million people worldwide due to travel and transmission. Without treatment, the protozoan infection progresses to the chronic phase, manifesting as a dilated cardiomyopathy subtype called Chagas cardiomyopathy, which is characterized by ventricular arrhythmias, fibrosis, and eventually heart failure [35]. Tatiana Araújo Silva et al. [36] utilized in vitro human cardiac fibroblast models and a mouse model of chronic CD to support the hypothesis that CD-caused parasite antigens or their secreted factors act as paracrine factors, leading to the excessive accumulation of extracellular matrix in cardiac fibroblasts and subsequent cardiac fibrosis in CD. This study demonstrated that pirfenidone, an inhibitor of transforming growth factor-beta (TGF-β) and platelet-derived growth factor, reduced collagen deposition in the mouse hearts and proposed pirfenidone as a novel approach to fibrosis therapy in CD. Pirfenidone is FDA-approved for the treatment of idiopathic pulmonary fibrosis, and its antifibrotic activity was reported in various animal models of cardiac disease [37].

Cirrhotic cardiomyopathy (CCM), characterized by progressive systolic and diastolic dysfunction, along with structural heart abnormalities, including fibrosis, develops in up to 50% of patients with liver cirrhosis that may progress to symptomatic HF [38]. Notably, liver transplantation significantly improves or reverses CCM and associated cardiac fibrosis [39]. Moritz Uhlig et al. [40] investigated the development and progression of CCM in a Sprague–Dawley rat liver injury model induced by bile duct ligation (BDL), aiming to advance our understanding of the impact of liver cirrhosis on cardiac function and structure in the first longitudinal study over a prolonged period. The author reported that an isolated fibrotic remodeling of left ventricular myocardium appeared only 8 weeks post-BDL-induced liver injury, accompanied by macrophage infiltration (assessed by increased number of CD68-positive cells in cardiac sections) and systemic inflammation (assessed by a tumor necrosis factor alpha—TNFα serum level). However, it was not associated with other typical signs of cardiomyopathy, such as significant functional impairment and the differential expression of the cardiomyopathy markers in the left ventricle. These results highlight the time-dependent and complex nature of CCM development and the importance of careful consideration in selecting the animal model that appropriately mimics the pathophysiology of the human disease.

Valosin-Containing Proteins (VCPs, ATPase-associated) play a complex and critical role in the heart, with their function depending on context and cellular conditions. The groundbreaking study by Hongyu Qiu et al. [41] represents the first generation and implication of a cardiac-specific knockout (KO) mouse model of VCPs, defining their essential role in heart development and in maintaining physiological function under unstressed conditions. It further reveals the distinct effects of VCPs on the mammalian target of rapamycin complex 1 and 2 (mTORC1 and mTORC2) signaling during cardiac compensatory stages. Importantly, the study identifies a novel mechanism of HF associated with VCP deficiency, characterized by dephosphorylation of p-AKT473, a key downstream target of mTOR2. Notably, this study identified a previously unrecognized role of cardiomyocyte VCPs in regulating protein phosphatase 1 (PP1) expression and nuclear translocation in the heart, establishing a mechanistic link between increased PP1 activity and reduced p-AKT473 levels in the old VCP KO mouse heart. Overall, this work provides new insights into the essential role of VCPs in cardiac biology, broadens the understanding of their regulatory influence on cellular signaling pathways, and highlights potential mechanisms underlying HF. These findings advance the knowledge of VCP function in the heart and may inform the development of targeted therapeutic strategies for VCP-related cardiac diseases.

Overall, this Special Issue, focuses on mechanisms and pathophysiology of myocardial disease and their comorbidities, highlighting the importance of pre-clinical and clinical research, which may represent a basis for innovative strategies for the prevention and treatment of myocardial disease to enhance life quality and promote longevity.

## Data Availability

Not applicable.

## Abbreviations

The following abbreviations are used in this manuscript:

HF	Heart Failure
ARNI	Angiotensin Receptor-Neprilysin Inhibitor
AV	Aortic Valve
HIF1A	Hypoxia-Induced Factor 1 Alpha
PBMCs	Peripheral Blood Mononuclear Cells
EndoMT	Endothelial-to-Mesenchymal Transition
ECs	Endothelial Cells
CD	Chagas Disease
TGF-β	Transforming Growth Factor-Beta
CCM	Cirrhotic Cardiomyopathy
BDL	Bile Duct Ligation
VCP	Valosin-Containing Protein
KO	Knockout
mTORC1	Mammalian Target of Rapamycin Complex 1
mTORC2	Mammalian Target of Rapamycin Complex 2
PP1	Protein Phosphate 1
